# Family Health Days program contributions in vaccination of unreached and under-immunized children during routine vaccinations in Uganda

**DOI:** 10.1371/journal.pone.0218239

**Published:** 2020-01-17

**Authors:** Ezekiel Mupere, Harriet M. Babikako, Violet Okaba-Kayom, Robert B. Mutyaba, Milton Nasiero Mwisaka, Emmanuel Tenywa, Albert Lule, Jane Ruth Aceng, Flavia Mpanga-Kaggwa, David Matseketse, Eresso Aga

**Affiliations:** 1 Department of Paediatrics & Child Health College of Health Sciences, Makerere University, Kampala, Uganda; 2 Research and Data System Center, Child and Family Foundation Uganda, Kampala, Uganda; 3 Child Health and Development Center College of Health Sciences, Makerere University, Kampala, Uganda; 4 Information Systems and Programming, RBMTM Systems Consult Limited, Kampala, Uganda; 5 Social Work Unit, Jinja Regional Referral Hospital Ministry of Health, Jinja, Uganda; 6 Paediatrics, Jinja Regional Referral Hospital Ministry of Health, Jinja, Uganda; 7 Nutrition Unit, Ministry of Health, Kampala, Uganda; 8 Minister for Health, Ministry of Health, Kampala, Uganda; 9 Health Office, UNICEF Uganda Country Office, Kampala, Uganda; 10 Health Manager, UNICEF Jordan Country Office, Amman, Jordan; The University of Warwick, UNITED KINGDOM

## Abstract

**Background:**

We explored the contributions of the Family Health Days (FHDs) concept, which was developed by the Uganda Ministry of Health (MOH) and UNICEF as a supplementary quarterly outreach program in addition to strengthening the routine expanded program for immunization (EPI), with the aim to increase coverage, through improved access to the unimmunized or unreached and under-immunized children under 5 years.

**Method:**

A cross-sectional descriptive study of the Uganda MOH, Health Management Information Systems (HMIS) and UNICEF in house FHDs data was conducted covering six quarterly implementations of the program between April 2012 and December 2013. The FHDs program was implemented in 31 priority districts with low routine vaccination coverage from seven sub-regions in Uganda in a phased manner using places of worship for service delivery.

**Results:**

During the six rounds of FHDs in the 31 districts, a total of 178,709 and 191,223 children received measles and Diphtheria-Pertussis-Tetanus (DPT3) vaccinations, respectively. The FHDs’ contributions were 126% and 144% for measles and 103% and 122% for DPT3 in 2012 and 2013, respectively of the estimated unreached annual target populations. All implementing sub-regions after two rounds in 2012 attained over and above the desired target for DPT3 (85%) and measles (90%). The same was true in 2013 after four rounds, except for Karamoja and West Nile sub-regions, where in some districts a substantial proportion of children remained unimmunized. The administrative data for both DPT3 and measles immunization showed prominent and noticeable increase in coverage trend in FHDS regions for the months when the program was implemented.

**Conclusion:**

The FHDs program improved vaccination equity by reaching the unreached and hard-to-reach children and bridging the gap in immunization coverage, and fast tracking the achievement of targets recommended by the Global Vaccine Action Plan (GVAP) for measles and DPT3 (85% and 90% respectively) in implementing sub-regions and districts. The FHDs is an innovative program to supplement routine immunizations designed to reach the unreached and under immunized children.

## Background

Immunization is a proven strategy for reducing morbidity and mortality among women and children in Africa [1]. Substantial investment has been made in the past three decades to establish and maintain national Routine Immunization (RI) systems [2–4] and progress has been remarkable in infrastructure establishment to support vaccination procurement and delivery including campaigns with Child Health Days (CHDs) and national immunizations to increase coverage rates more rapidly [5].

Uganda has specifically accomplished greatly on enhancing its routine immunization (RI) system function [6]. For example, a) Uganda developed district-level strategies for improvement with the World Health Organization (WHO) and partners [7], (b) conducted evaluation studies [8, 9], (c) developed a training manual [6] for operational-level staff that incorporates the Reaching Every District (RED) strategy [10, 11], (d) launched the RED in 2003, and (e) developed numerous strategies to sustain immunization rates when rates are high and improve rates when rates are low as outlined by the Uganda National Expanded Program on Immunization (UNEPI) multi-year plan [12].

Despite Uganda’s achievements in improving the RI system, with success in reaching high levels of Diphtheria-Pertussis-Tetanus third dose (DPT3) coverage, improving rapidly from 9% in 1980 to a high of 82% in 2011, the coverage was uneven or varied greatly from one district to another and equity in access to vaccination remained a challenge in several districts. A large number of children were unimmunized/or unreached and under-immunized. In 2010, Uganda was one of the five countries in Africa that contributed to the 3.99 million infants who did not receive all three doses of DPT containing vaccine [13]. Findings of the Uganda Demographic Health Survey (UDHS) 2011 revealed also the less than optimal results for immunization. According to the survey, only 52% of children aged between 12–23 months were fully vaccinated [14]. Further, other programs designed to improve immunization coverage, such as the Child Health Days (CHDs), the Reach Every Child approach (REC), were not quite effective and showed coverage challenges. Overall, vaccination coverage stagnated and demonstrated a leveling out of the performance, particularly in districts with hard to reach population and high child mortality rates.

Considering the immunization service delivery challenges such as limited knowledge and awareness about the benefits of immunization; family hierarchy and weak male involvement in child immunization; opportunity cost associated with service uptake; religious and cultural concerns in some areas in the country; and coverage level stagnation in 2012 [14–18], MOH, UNICEF and partners decided to strengthen service delivery and developed the concept of Family Health Days (FHDS) and its implementation guidelines. The FHDs explored the use of places of worship (POWs) as entry point to communities and points of services delivery for vaccinations to the populace of children 0–59 months that could have missed routine and other static services at health facilities. The services were delivered at churches and mosques on prayer days to complement and extend the reach of facility-based and other outreach health services.

The FHDs were held quarterly in January, April, July and October through mass community mobilization for health services uptake based on the experience CHD plus. Regular supportive supervision for microplanning, supply and cold chain management, implementation and monitoring was done jointly by Ministry of health and local government officials as well as UNICEF staff in Kampala as the three UNICEF field offices in Acholi, Karamoja and Western regions. Technical working groups (TWGs) for planning supply chain managements, promotion and social mobilization as well as monitoring and evaluation were formed and convened on a regular basis to assess challenges, lessons learned, share good practices for course correction and adjustments as needed. The TWGs reported directly to a steering committee consisting of senior officials from MOH and UNICEF for overall strategic guidance. The first two rounds of FHDs were implemented in July and October of 2012 in 20 UNICEF priority districts. By December 2013, the implementation had been expanded to 31 districts in seven sub-regions in the country. A systematic analysis of the FHDs data at UNICEF Uganda and HMIS data at the MOH was conducted. In this paper, we report the results of this analysis that evaluated the contributions of the FHDs implementation in improving access to the unimmunized or unreached and under-immunized children, and fast-tracking of the Expanded Program in Immunization (EPI) indicators in poorly performing districts in the country.

## Methods

### Design

A cross-sectional descriptive analysis of UNICEF in house FHDs and the Uganda MOH health management information systems (HMIS) data from April 2012 to December 2013 was performed to evaluate the effectiveness of the FHDs program in vaccination of the unreached children by the routine EPI program. The monitoring and evaluation staff for UNICEF systematically collected anonymized aggregated vaccination data from all the 31 implementing districts in seven sub-regions parallel to the routine vaccination data transmission by the districts through the HMIS at MOH. The HMIS data is transmitted routinely as anonymized data by all districts in the country into the MOH data management system.

### Ethical issues

The study protocol was reviewed, approved, and waiver of consent to use UNICEF and HMIS data was provided by the School of Biomedical Sciences Research Ethical Committee at the College of Health Sciences, Makerere University. Permission to use the HMIS data was obtained at the Ministry of Health through the office of the Director General. All UNICEF program and HMIS data were fully anonymized prior to access and use in the analysis.

### Implementation and monitoring of FHDs

FHDs program was implemented in 31 priority districts with low routine vaccination coverage or with high infant mortality rates from seven sub-regions in Uganda in a phased manner, and by end of 2013 six rounds had been implemented in the months of July and October 2012; and Jan/Feb, April, July, and October of 2013 (S1 Fig). The first two FHDs rounds were implemented in the months of July and October 2012, and 20 districts were involved including Abim, Kotido, Kaabong, Amudat, Nakapiripirit, Napak and Moroto in Karamoja sub-region; Lamwo, Kitgum, Pader, Agago, Gulu, Nwoya and Amuru in Acholi sub-region; and Mubende, Kyegegwa, Kyenjojo, Kabarole, Ntoroko and Bundibugyo in Western sub-region. When the third and fourth rounds in January/February and April 2013 were planned and conducted, implementation was scaled-up to cover eight additional districts of Wakiso, Lwengo, Bukomansimbi, Mukono, Kalungu, and Buikwe in Central; and Yumbe and Arua in West Nile sub-regions (S1 Fig). In July 2013, Kampala district was added to the implementing districts and in October 2013, FHDs implementation was expanded to Busoga sub-region to include Kamuli and Iganga districts; leading to a total of 31 FHDs implementing districts. Most of the beneficiary populations except in Kampala hosting the capital city were rural.

The services provided through the FHDs primarily targeted children under-five years of age (0–59 months) with EPI services; however, the package was expanded to improve health for pregnant, lactating and non-lactating women, as well as men. The existing national and district support functions for routine services were strengthened to support FHDs program including planning and coordination, resource leverage with partners, supplies and logistics, supervision and monitoring. The District Health Officers (DHOs) were responsible for the overall coordination and implementation of the FHDs in their respective district and the district health management teams supervised the activities with support of UNICEF Project Officers that provided feedback and capacity building to service providers.

Health care service delivery was provided by health care workers from public, private-not-for-profit, and private-for-profit facilities where possible within the catchment area after orientation and sensitization on the conduct of FHDs by the district health management team and UNICEF Project Officers. The service delivery for FHDs was conducted for a month in each district as extension of facility-based services at POWs on prayer days of Friday, Saturday, and Sunday and at community outreach post sites identified by community members for hard-to-reach or mobile populations. Health care workers were paid a safari-day-allowance for this extra effort.

The standard MOH HMIS forms and registers such as immunization tally sheets and child registers were used for data collection and reporting by the health care workers. Where appropriate HMIS were not available, standardized reporting forms were developed by UNICEF in conjunction with MOH for use during FHDs outreach activities. All data was transmitted by the district health teams to the Districts health information system 2 (DHIS 2) at the MOH’s national resource center. The key EPI indicators that were used for monitoring the FHDs included the number and percent of children <1 year that received DPT3 and measles vaccinations.

### Analysis

Contributions of the FHDs program was evaluated using the output EPI indicators for DPT3 and measles. Baseline indicator levels in 2011 for each sub-region and district were compared with set FHDs targets in 2014. The HMIS data at MOH, Annual Health Sector Performance Report (AHSPR) at MOH, and UNICEF summary data were used. For validation and to understand the contributions of the FHDs program, changes in indicators by 2014 for routine measles and DPT3 vaccination coverage in 26 FHDs districts were compared to coverage in 22 non-FHDs districts (S1 Fig).

To further understand whether the FHDs program made a difference with the EPI services offered in the implementing sub-region and districts, the extent of annual FHDs contributions to EPI annual FHDs indicators and contributions to the unreached populations missed during routine services were computed using the UNICEF In-house FHDs data. Proportions of FHDs contributions were computed according to the output FHDs indicators as were described in the FHDs guidelines, and to the unreached populations that were missed during routine services. Stratified analysis was used to compute FHDs contributions to the national and district EPI indicators by first computing marginal totals of the beneficiaries for each indicator as numerators and populations in each implementing district. The Uganda bureau of statistics (UBOS) 2012 and 2013 populations and proportions of demographic distributions [14] were used to estimate the denominator populations of interest according to the national and district populations.

Stratified analysis was also used at regional and district levels to compute FHDs contributions to the unreached populations missed during routine services. The district totals for each FHDs service were used as numerators and populations were aggregated into sub-region marginal totals according to the number of districts involved. The 31 implementing districts were aggregated into seven operational sub-regions of Acholi (7 districts), Karamoja (7 districts), Western (6 districts), Central (6 districts), West Nile (2 districts), Kampala (1 district) and Busoga (2 districts). The unreached populations as denominators for each FHDs service was estimated by subtracting off reached populations during routine service from the denominator populations of interest using UDHS 2011 [14] and UNEPI 2013 proportion estimates. The FHDs contributions towards the annual target in the unreached proportion were computed as proportions of the number of beneficiaries in the population of interest to the total unreached population in FHDs district, regional and national level.

## Results

### FHDs contributions in vaccination of children unreached by routine program

**Fig 1:**
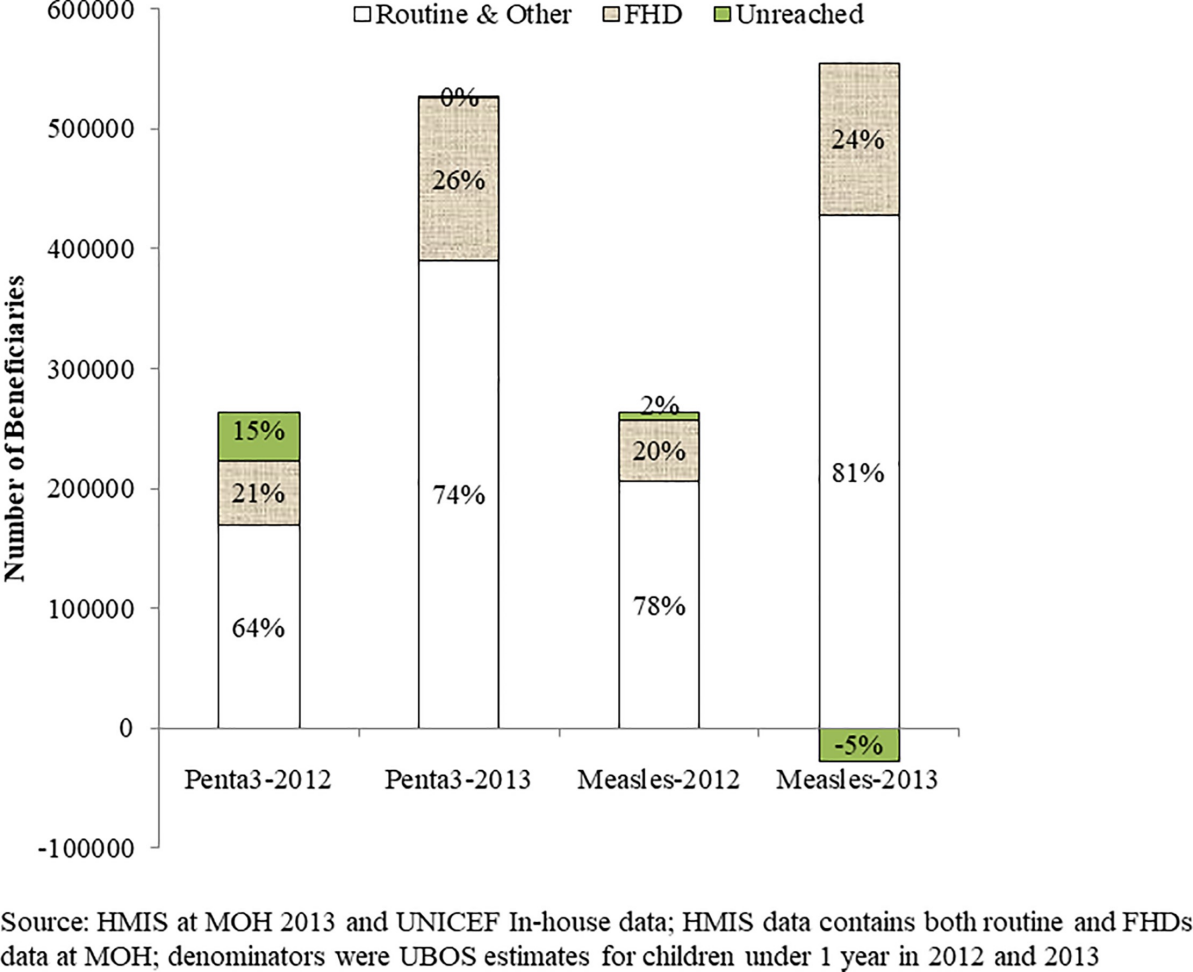
Annualized FHDs and routine contributions to measles and DPT3 vaccinations in 2012 and 2013 rounds in 31 implementing districts. Source: Health Management Information System (HMIS) at Ministry of Health (MOH) 2013 and UNICEF In-house data; HMIS data contains both routine and FHDS data at MOH; denominators were UBOS estimates for children under 1 year in 2012 and 2013.

Of the 178709 and 191223 children that received measles and DPT3 vaccinations, respectively during the six FHDs rounds in 31 implementing districts, the FHDs contributions were 126% and 144% for measles and 103% and 122% for DPT3 in 2012 and 2013, respectively of the estimated unreached annual target populations using UBOS population estimates. The FHDs program contributed in bridging the gap of unreached population. When HMIS data was evaluated, FHDs contributed 20% and 24% to the measles vaccination coverage in 2012 and in 2013, respectively. FHDs helped to reduce the unreached children significantly in 2012 and exceeded the eligible population by 5% in 2013. Similarly, FHDs contributed 21% and 26% to the DPT3 vaccination coverage in 2012 and 2013, respectively (Fig 1).

**Fig 2:**
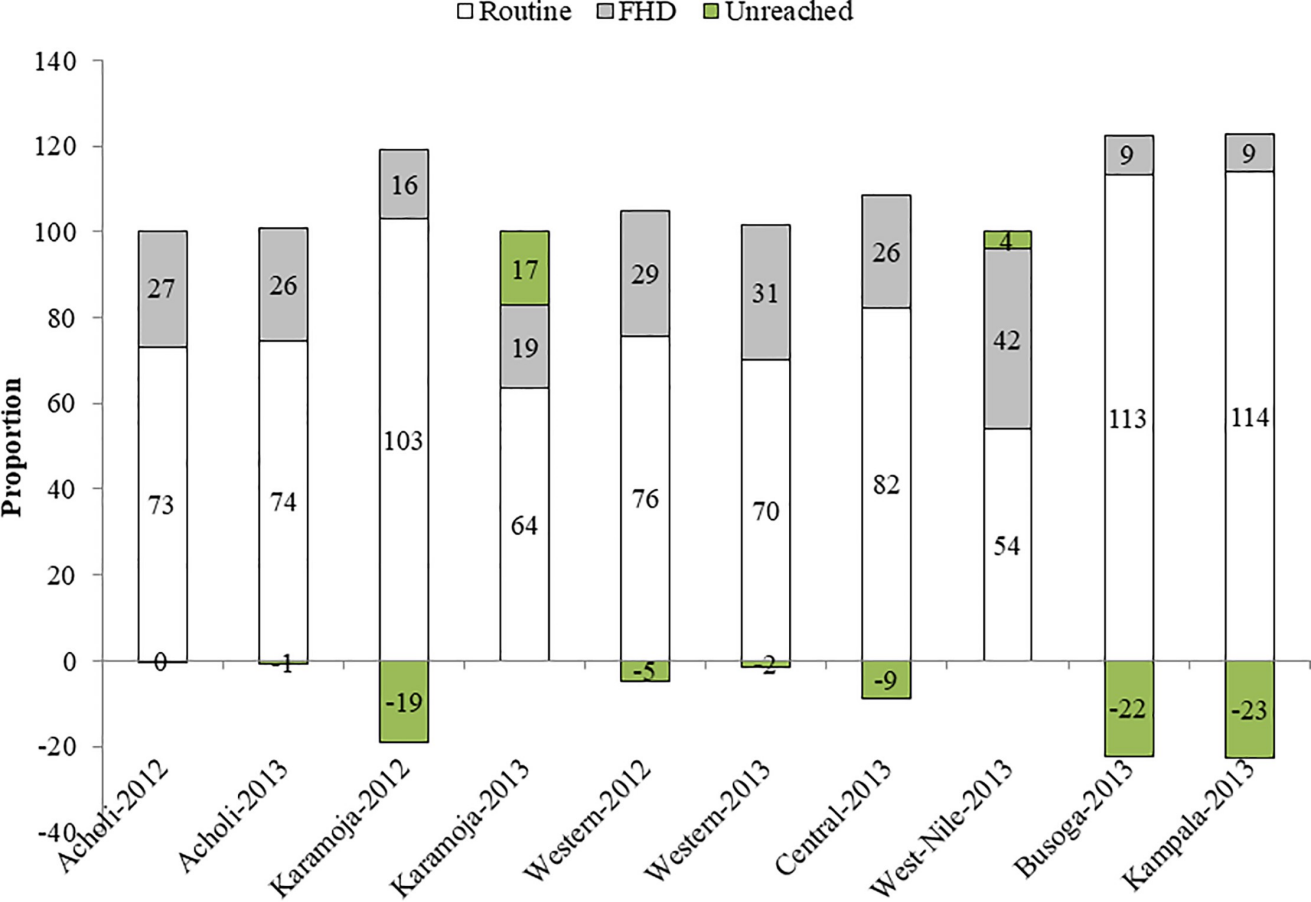
Annualized FHDs and routine contributions to measles vaccinations in 2012 and 2013 rounds in seven implementing sub-regions. Source: Health Management Information System (HMIS) at Ministry of Health (MOH) 2013 and UNICEF In-house data; HMIS data contains both routine and FHDs data at MOH; denominators were UBOS estimates for children under 1 year in 2012 and 2013.

**Fig 3:**
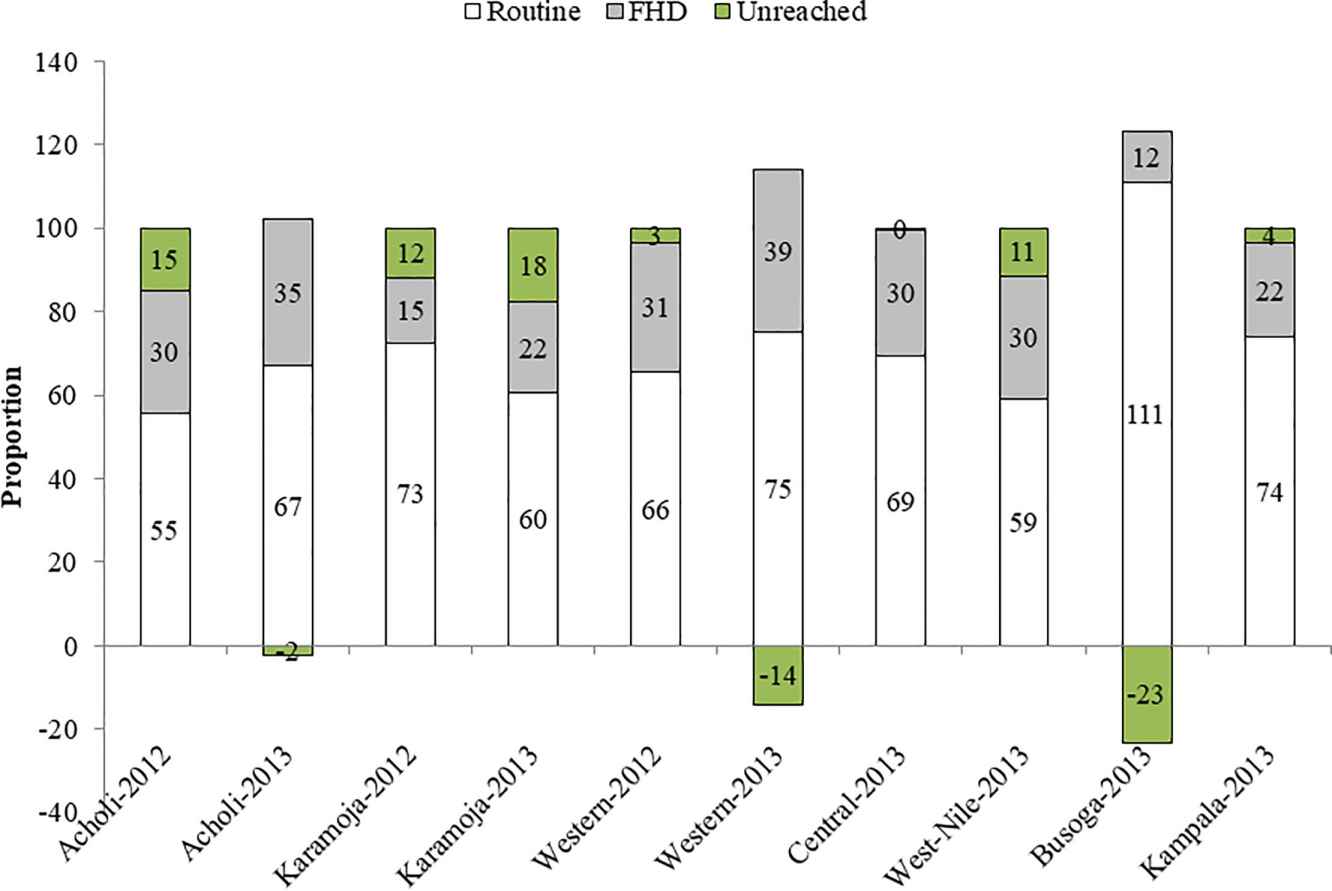
Annualized FHDs and routine contributions to DPT3 vaccinations in 2012 and 2013 rounds in seven implementing sub-regions. Source: Health Management Information System (HMIS) at Ministry of Health (MOH) 2013 and UNICEF In-house data; HMIS data contains both routine and FHDs data at MOH; denominators were UBOS estimates for children under 1 year in 2012 and 2013.

**Fig 4:**
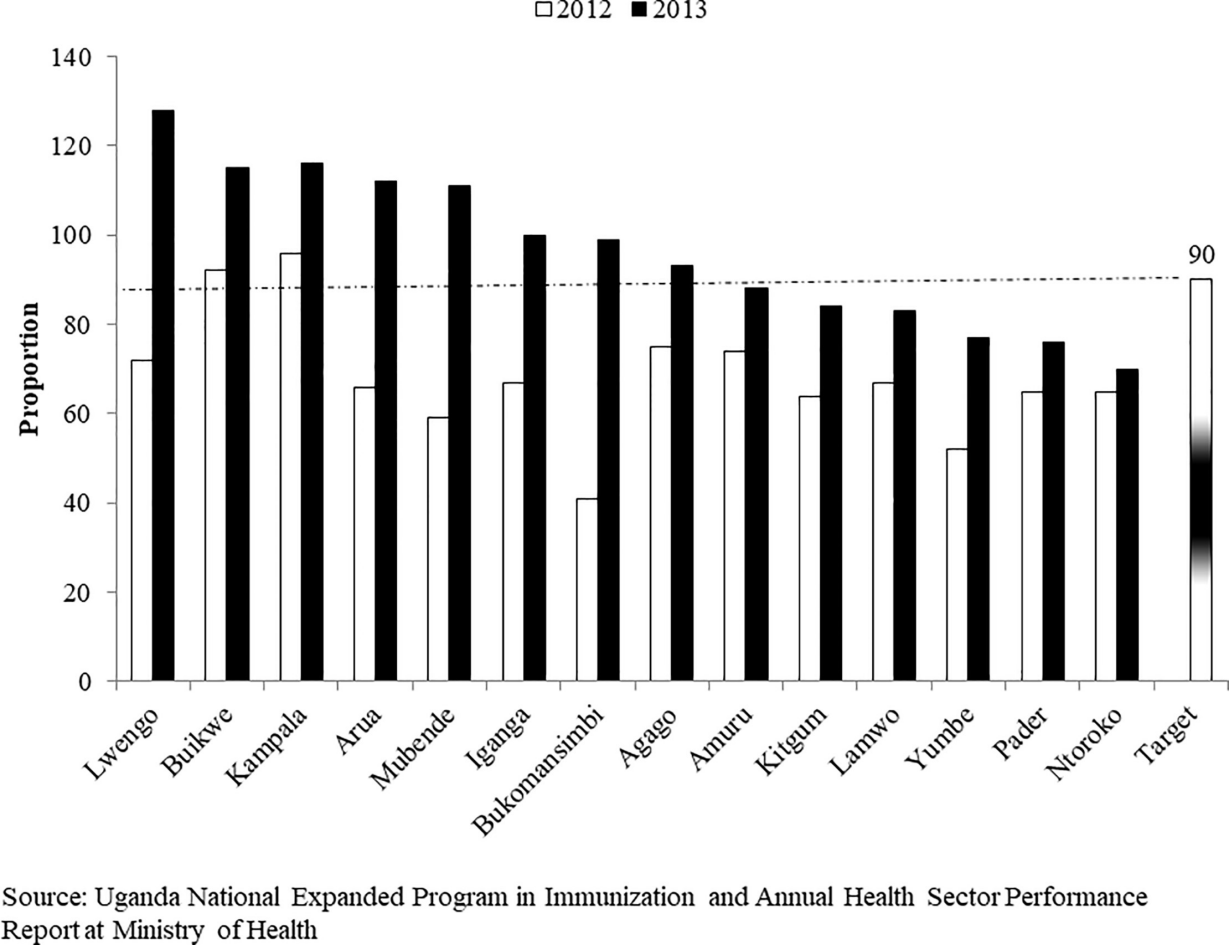
Trends of measles vaccination coverage for notable districts during FHDs implementation periods in 2012 and 2013. Source: UNEPI and Annual Health Sector Performance Report (AHSPR) at Ministry of Health.

**Fig 5:**
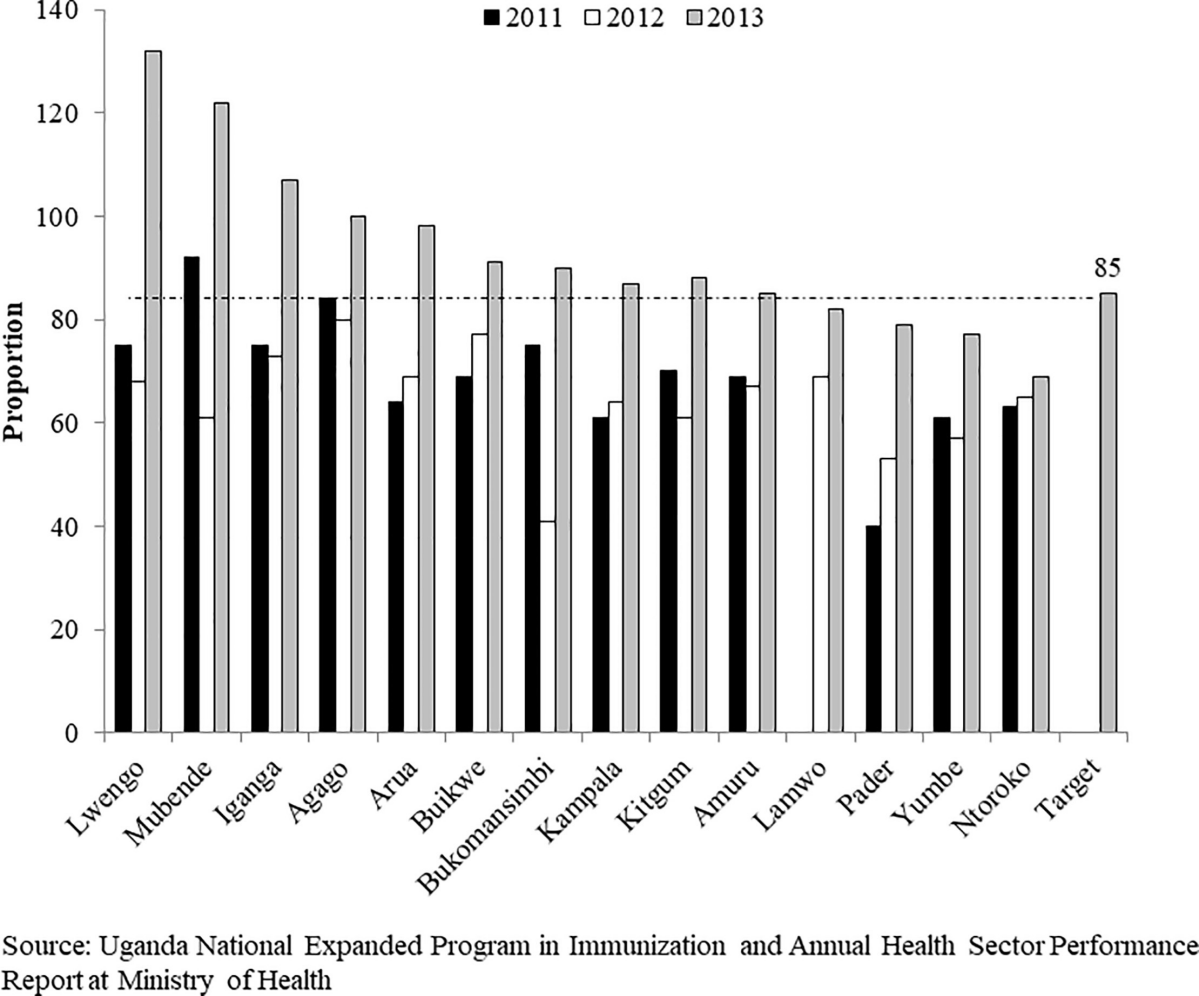
Trends of DPT3 vaccination coverage for notable districts during FHDs implementation periods in 2012 and 2013. Source: UNEPI and Annual Health Sector Performance Report (AHSPR) at Ministry of Health.

The FHDs program contributed in fast tracking the implementing sub-regions and districts to reach desired targets of 90% and 85% for measles and DPT3 coverage, respectively (Figs 2, 3, 4 and 5). There were dramatic sub-regional variations in FHDs contributions to measles vaccinations in 2012 and 2013 (Fig 2). All implementing sub-regions after two rounds in 2012 attained over and above the desired measles target of 90%. The same was true in 2013 after four rounds except Karamoja and West Nile sub-regions (Fig 2). The routine measles vaccination was optimal for Karamoja in 2012 and for Kampala and Busoga regions in 2013. The program contribution was even more remarkable when a sub-region had low baseline routine coverage such as West Nile at 59%. The eligible target populations were superseded in Karamoja and West Nile sub-regions in 2012 and in Kampala, Busoga, and Central sub-regions in 2013 (Fig 2).

The FHDs program contributed in fast tracking DPT3 vaccination in all implementing sub-regions to reach the desired target of 85% in 2012 and 2013 except Karamoja in 2013. The unreached population decreased substantially in Acholi, Western and Central sub-regions by 2013. The DPT3 coverage superseded the eligible target populations for Acholi, Western and Busoga sub-regions (Fig 3).

When immunization trends were evaluated by district, there were dramatic gains in measles coverage for the year 2013 in 14 districts. Of the 14 districts, 8 (57%) attained the desired target of 90% (Fig 4). The FHDs program contributed to an increase in measles vaccination coverage also for the twelve districts that already had a coverage above 90% during routine vaccination. Examples of districts that already had a good coverage through routine vaccination were: Nwoya in Acholi; Nakapiripirit and Napak in Karamoja; Kyegegwa in Western; Buikwe, Kampala, Mukono and Wakiso in Central; and Kamuli in Busoga sub-regions (S2 Fig).

There were remarkable gains in DPT3 coverage for the year 2013 in 14 districts that had vaccination coverage below the desired target of 85% (Fig 5). Of the 14 districts, 10 (71%) attained the desired target. The FHDs program contributed to an increase in the DPT3 vaccination for thirteen districts that already had high (85% and above) routine coverage. The districts that had good routine DPT3 coverage were Gulu and Nwoya in Acholi, Kotido, Nakapiripirit, and Napak in Karamoja, Kabarole in Western, Mukono in Central, Kamuli in Busoga (S3 Fig).

**Fig 6:**
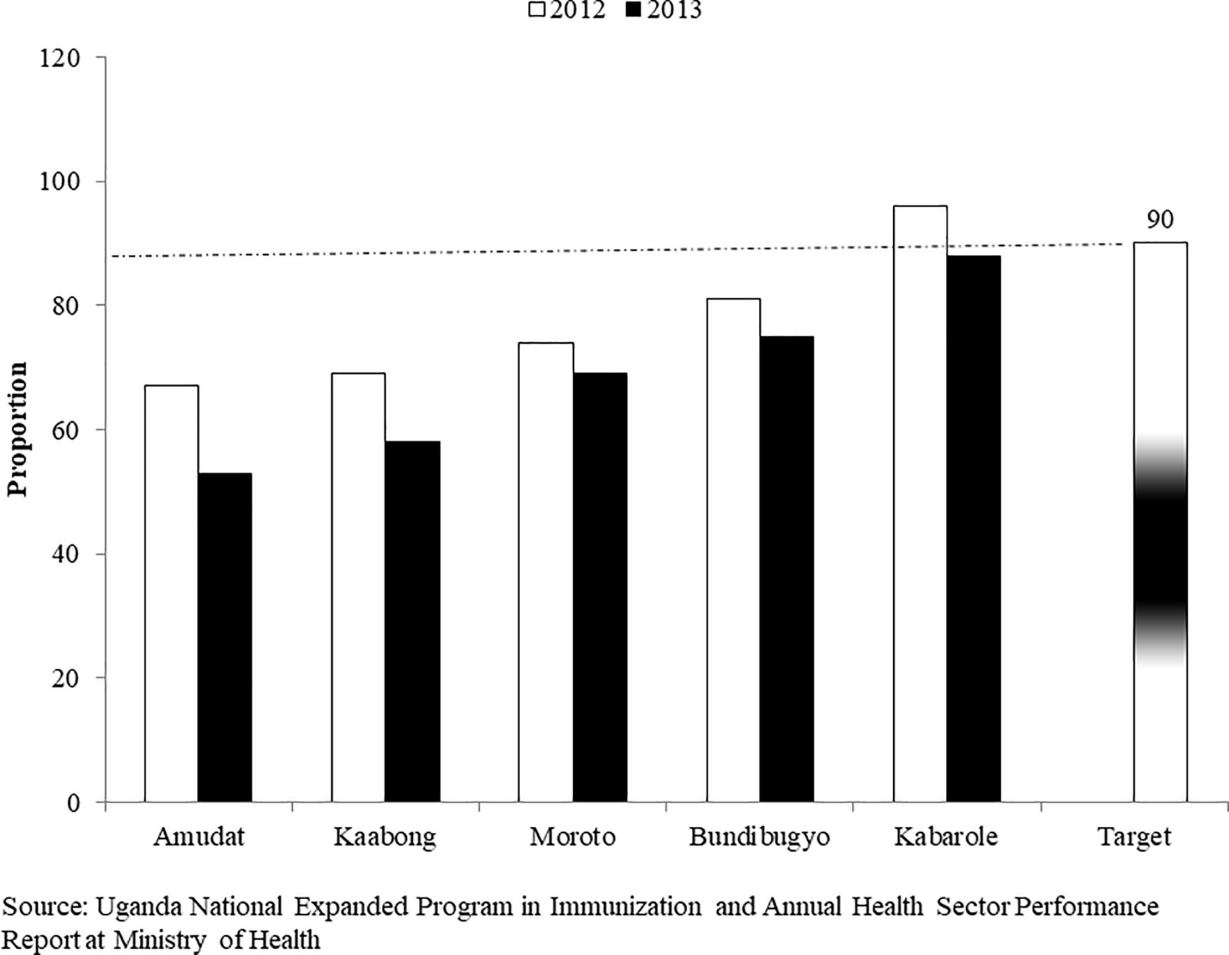
Measles vaccination coverage trends for districts with poor performance during FHDs implementation in 2012 and 2013. Source: UNEPI and Annual Health Sector Performance Report (AHSPR) at MOH.

**Fig 7:**
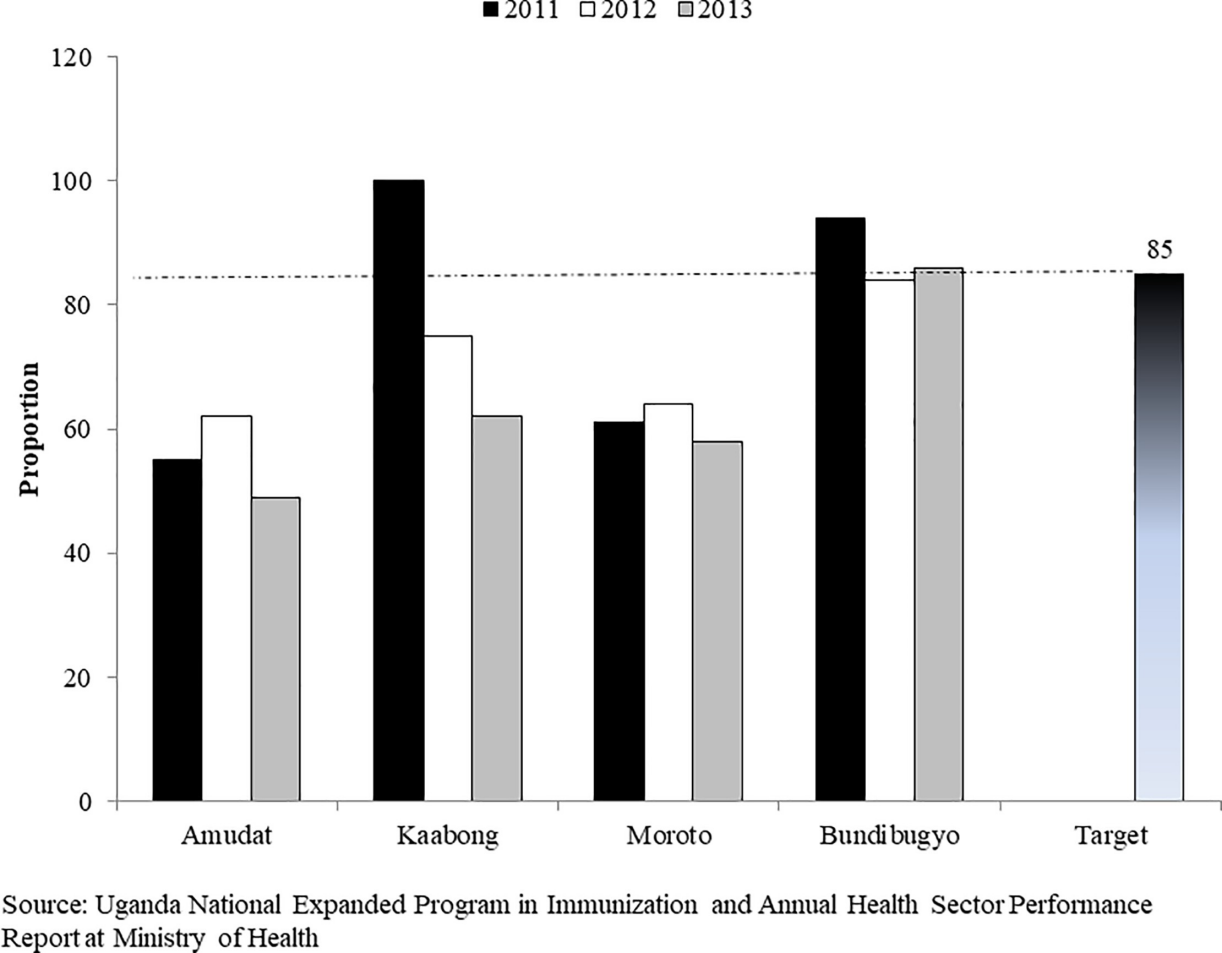
DPT3 vaccination trends for districts with poor performance during FHDs implementation, 2011–2013. Source: UNEPI and Annual Health Sector Performance Report (AHSPR) at MOH.

Despite implementation of six rounds in certain districts of Amudat, Moroto, Kaabong, Bundibugyo and Kabarole, there were minimal changes in Measles and DPT3 vaccination coverage in 2012 and 2013 (Figs 6 and 7). Moreover, the routine coverage indicators were below the set targets of 90% for measles and 85% for DPT3 in Amudat, Moroto, and Kaabong district prior to FHDs implementation. FHDs contributions to measles and DPT3 vaccinations were lowest in Moroto and highest in Kaabong.

### Where are the unimmunized children in the FHDs implementing districts?

A substantial proportion of children remained unimmunized during FHDs implementation in certain districts of Kaabong (18% for DPT3 versus 24% for measles) in Karamoja sub-region, Yumbe (17% for DPT3 versus 21% for measles) in West Nile sub-region, and Kampala (17% for DPT3) in Central sub-region (Table 1). Modest burden was found in Amudat (7% for DPT3 versus 8% for measles), Moroto (7% for DPT3 versus 6% for measles), Pader (6% for DPT3 versus 8% for measles) and Wakiso (6% for DPT3) districts.

**Table 1:** Distribution of un-immunized children in FHDs implementing districts.

Districts	DPT3		Measles	
	Numbers	%	Numbers	%
Kaabong	7198	18	7857	24
Yumbe	6645	17	6823	21
Kampala	6520	17	-	-
Amudat	2708	7	2493	8
Moroto	2697	7	2034	6
Pader	2340	6	2677	8
Wakiso	2266	6	-	-
Kitgum	1235	3	1759	5
Bundibugyo	847	2	2456	7
Kabarole	-	-	1537	5
Lamwo	1421	4	1358	4
Ntoroko	1237	3	1189	4
Buikwe	1614	4	-	-
Amuru	1164	3	951	3
Agago	260	1	1211	4
Kotido	-	0	308	1
Bukomansimbi	528	1	-	0
Kalungu	155	0	263	1
Arua	152	0	-	-
Mukono	12	0	-	-
**Total**	**39,000**	**100**	**32,916**	**100**

## Discussion

We conducted a cross-sectional systematic analysis of the FHDs data at UNICEF Uganda and the HMIS data at MOH to evaluate the contribution of the FHDs implementation in improving access to the unimmunized or unreached and under-immunized children in 31 districts in seven sub-regions in the country. We found that the FHDs program contributed in improving access to vaccination of children unreached by the routine program even when only two rounds were conducted in both normal and hard to reach sub-regions and districts in the country. The program contributed in fast tracking districts or sub-regions to reach desired targets of 90% and 85% for measles and DPT3 coverage, respectively. However, the fast tracking or dramatic contributions by the FHDs program differed by sub-regions and districts. It was marked in districts from West Nile, Western and Acholi sub-regions and modest in Karamoja and Kampala in 2013. The program superseded eligible target populations in Acholi, Western and Busoga sub-regions. Districts that had low vaccination coverage prior to the program had dramatic gains in coverage. However, Amudat, Moroto and Kaabong districts in Karamajo and Bundibugyo and Kabarole districts in Western sub-regions had minimal changes in vaccination despite six rounds of the FHDs program.

Our findings suggest that routine immunization supported with opportunities of regular and periodic intensified vaccination services such as FHDs improves vaccination coverage in areas that are hard to reach; areas where coverage is low and where dropout levels are high. In this review, we found dramatic gains in coverage in sub-regions and districts that had low vaccination coverage prior to the program. The FHDs contribution in improving access to vaccinations beyond the target populations would be explained by for major characteristics of the program.1) The first is the use of faith-based organizations (FBOs) as entry point to communities to offer places of worship (POW) to deliver vaccination services. POWs have a wide geographical coverage; have close proximity to users to easily access services and are physical structures that already exist to offer services without extra investment. Further, most Ugandans are religious and are affiliated to churches and mosques. The religious leaders at POWs are trusted and respected by community members. This strength was tapped for socio-mobilization into the FHDs program to have increased service uptake. The religious leaders mobilized the congregations for FHDs services. When the religious leaders sanctioned FHDs services, this encouraged acceptance of immunization services. The strengths of FBOs in socio-mobilization and in promotion of health services have been demonstrated in several previous studies and programs [19, 20].

2) The second explanation for the pronounced success of FHDs in improving vaccination coverage could have been the integrated nature of the service delivery on weekends. Offering services on weekends offered families as a unit a better opportunity to bring children for child health to service points because it fell outside of official working days. 3) The third contribution was that the FHDs services encouraged male involvement. During the FHDs implementation, men were offered opportunity to have their blood pressures taken as mothers and the children received antenatal care and child health services, respectively. This led to increased male involvement and had a remarkable impact on mobilization of beneficiaries. 4) The fourth reason for the programs impact explanation may be explained by the components of the program, which were aimed at strengthening the overall immunization system, such as microplanning, social mobilization, supply chain management, data quality improvement, support for cold chain status assessment and maintenance as well as regular field visits and supportive supervision by UNICEF and MOH officers.

In this review, we found Amudat, Moroto and Kaabong districts in Karamajo and Bundibugyo and Kabarole districts in Western sub-regions to have had minimal changes in vaccination despite a large population of the unreached prior and six rounds of the FHDs program. Some of the possible reasons to explain could have been 1) gaps in the structures of the health systems in the districts, low morale and attitude by the district health teams (DHTs); 2) poor data management system to collect and transmit through HMIS; 3) inadequate staffing, few number of health facilities, and POWs; 4) the harsh population environment with low socio-economic and education status; 5) and poor communication and mobilization of beneficiaries for routine and FHDs services. The review found that the support supervision and monitoring frequency by UNICEF Program Officers and MOH Central supervisors was limited in some of these districts particularly Moroto and Kaabong to offer the necessary mentorship and technical support because of poor road system.

The possible explanations for the persistent burden of the unimmunized children in certain districts that implemented FHDs could be due to the sub-region or the individual district’s geographical settings, structural and health system performance functions. Kaabong is a large district bordering Kenya and Southern Sudan; therefore, constantly receives an influx of foreign populations. The district has hard to reach villages on hills and in valleys, and a large number of mobile cattle keeping populations. The health facilities and district staff were and are inadequate to serve the existing population. Yumbe is also a district on the border with Southern Sudan, commonly receiving refugee population coupled with limited facilities and staffing issues. The generally low education level in these districts may be responsible for the poor attitude and low uptake of immunization services. Kampala district experiences unique challenges of a large metropolitan city with large numbers of urban informal settlements and new populations with limited access to public health services. The populations are transient with differing health seeking behaviors. The urbanized populations with most caretakers involved in informal jobs could hamper health care seeking. The caretakers fail to apportion time to take the children for immunization.

In conclusion, the FHDs program contributed in bridging the gap of unreached and under immunized children for measles and DPT3 and in fast tracking the implementing sub-regions and districts to reached desired targets of 90% and 85% for both antigens, respectively. The FHDs program improved routine vaccination uptake for both DPT3 and measles in the implementing sub-regions. The FHDs is an innovative program to supplement routine immunizations in accessing unreached and under immunized children. However, in the face of strong routine coverage, the FHDs program is less pronounced. The overall program success indicates that interventions aimed at strengthening health systems and use of existing community entry points such as POWs, to deliver services are strategic in removing access barrier, improve acceptability of services and increase uptake of critical preventive health services. However, the cost-effectiveness and sustainability of such interventions may need to be explored.

## Supporting information

S1 Fig Districts implemented Family Health Days. (TIF)

S2 Fig Annualized measles vaccination coverage for 2012 and 2013 in high performing districts. Source: Health Management Information System (HMIS) at Ministry of Health (MOH) 2012 and 2013; HMIS data contains both routine and FHDs data at MOH; denominators were UBOS estimates for children under 1 year in 2012 and 2013
(TIF)

S3 Fig Annualized DPT3 vaccination coverage for 2012 and 2013 in high performing districts. Source: Health Management Information System (HMIS) at Ministry of Health (MOH) 2012 and 2013; HMIS data contains both routine and FHDs data at MOH; denominators were UBOS estimates for children under 1 year in 2012 and 2013.
(TIF)
