# Supplementary figures and images for: Family Health Days program contributions in vaccination of unreached and under-immunized children during routine vaccinations in Uganda
Source: PLoS One. 2020 Jan 17;15(1):e0218239. doi: 10.1371/journal.pone.0218239 (PMC6968838; doi:10.1371/journal.pone.0218239)

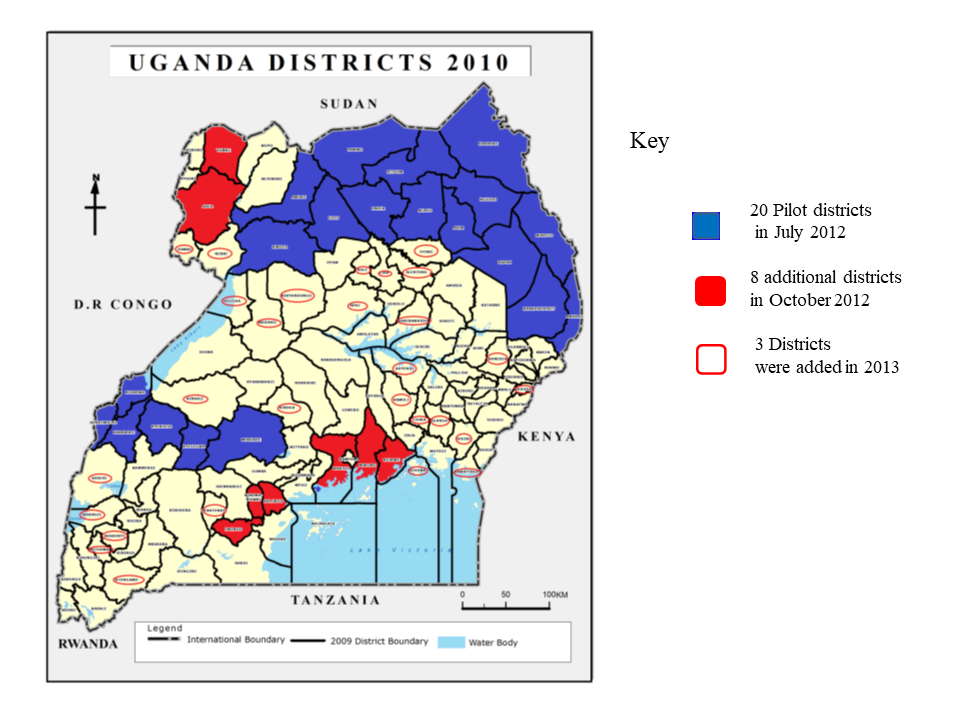

Supplement: S1 Fig — (TIF) [file pone.0218239.s001.tif]

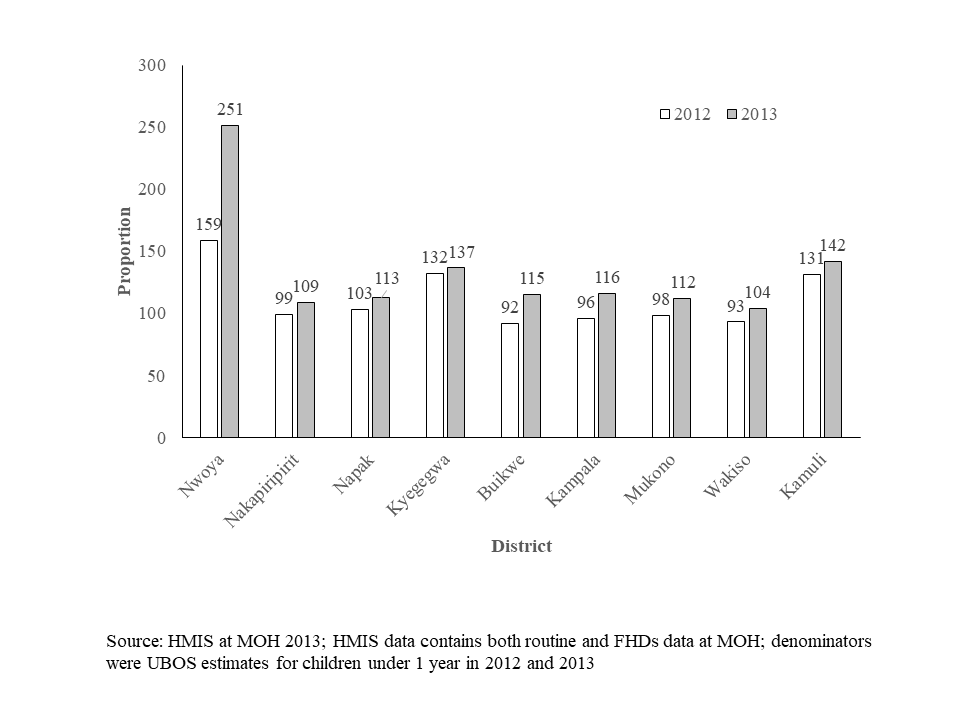

Supplement: S2 Fig — Source: Health Management Information System (HMIS) at Ministry of Health (MOH) 2012 and 2013; HMIS data contains both routine and FHDs data at MOH; denominators were UBOS estimates for children under 1 year in 2012 and 2013 (TIF) [file pone.0218239.s002.tif]

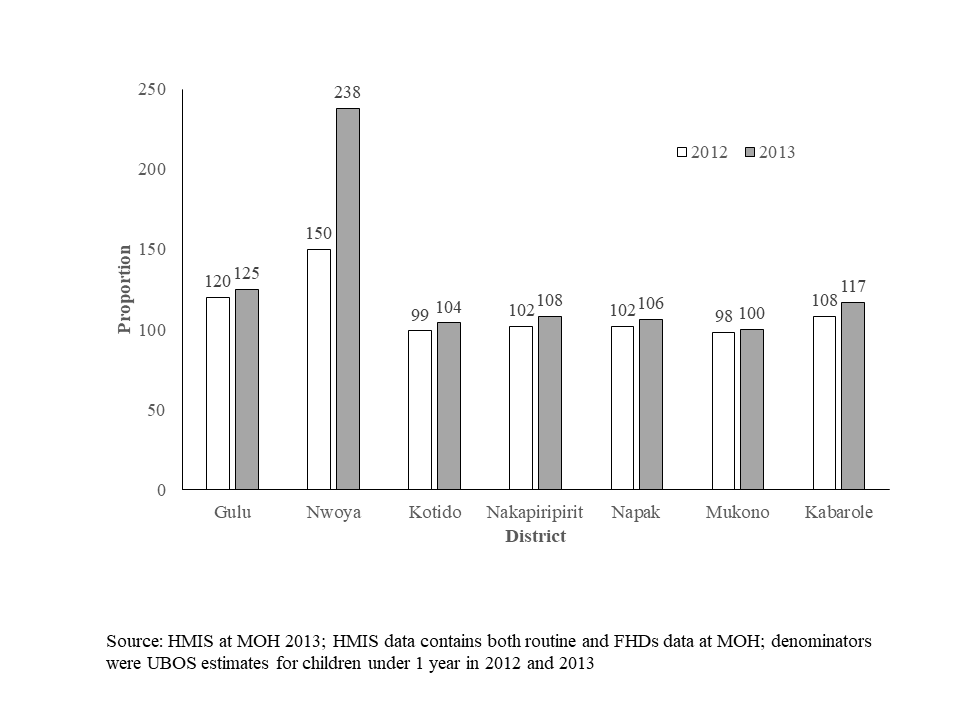

Supplement: S3 Fig — Source: Health Management Information System (HMIS) at Ministry of Health (MOH) 2012 and 2013; HMIS data contains both routine and FHDs data at MOH; denominators were UBOS estimates for children under 1 year in 2012 and 2013. (TIF) [file pone.0218239.s003.tif]
